# Mechanism of Inhibition of Ebola Virus RNA-Dependent RNA Polymerase by Remdesivir

**DOI:** 10.3390/v11040326

**Published:** 2019-04-04

**Authors:** Egor P. Tchesnokov, Joy Y. Feng, Danielle P. Porter, Matthias Götte

**Affiliations:** 1 Department of Medical Microbiology and Immunology, University of Alberta, Edmonton, AB T6G 2E1, Canada; tchesnok@ualberta.ca; 2 Li Ka Shing Institute of Virology at University of Alberta, Edmonton, AB T6G 2E1, Canada; 3 Gilead Sciences, Inc., Foster City, CA 94404, USA; Joy.Feng@gilead.com (J.Y.F.); Danielle.Porter@gilead.com (D.P.P.)

**Keywords:** Ebola virus, respiratory syncytial virus, RNA polymerase, RdRp, remdesivir, GS-5734, delayed chain termination

## Abstract

Remdesivir (GS-5734) is a 1′-cyano-substituted adenosine nucleotide analogue prodrug that shows broad-spectrum antiviral activity against several RNA viruses. This compound is currently under clinical development for the treatment of Ebola virus disease (EVD). While antiviral effects have been demonstrated in cell culture and in non-human primates, the mechanism of action of Ebola virus (EBOV) inhibition for remdesivir remains to be fully elucidated. The EBOV RNA-dependent RNA polymerase (RdRp) complex was recently expressed and purified, enabling biochemical studies with the relevant triphosphate (TP) form of remdesivir and its presumptive target. In this study, we confirmed that remdesivir-TP is able to compete for incorporation with adenosine triphosphate (ATP). Enzyme kinetics revealed that EBOV RdRp and respiratory syncytial virus (RSV) RdRp incorporate ATP and remdesivir-TP with similar efficiencies. The selectivity of ATP against remdesivir-TP is ~4 for EBOV RdRp and ~3 for RSV RdRp. In contrast, purified human mitochondrial RNA polymerase (h-mtRNAP) effectively discriminates against remdesivir-TP with a selectivity value of ~500-fold. For EBOV RdRp, the incorporated inhibitor at position i does not affect the ensuing nucleotide incorporation event at position i+1. For RSV RdRp, we measured a ~6-fold inhibition at position i+1 although RNA synthesis was not terminated. Chain termination was in both cases delayed and was seen predominantly at position i+5. This pattern is specific to remdesivir-TP and its 1′-cyano modification. Compounds with modifications at the 2′-position show different patterns of inhibition. While 2′-C-methyl-ATP is not incorporated, ara-ATP acts as a non-obligate chain terminator and prevents nucleotide incorporation at position i+1. Taken together, our biochemical data indicate that the major contribution to EBOV RNA synthesis inhibition by remdesivir can be ascribed to delayed chain termination. The long distance of five residues between the incorporated nucleotide analogue and its inhibitory effect warrant further investigation.

## 1. Introduction

Infection with negative-sense RNA viruses is associated with a broad spectrum of human diseases. Influenza and respiratory syncytial virus (RSV) are recognized as widespread global pathogens, while others, such as Ebola virus (EBOV), Lassa virus (LASV), or Crimean–Congo hemorrhagic fever virus (CCHFV) are known for sporadic outbreaks, their high epidemic potential, and their high mortality rates [1]. Negative-sense RNA viruses are classified into segmented and non-segmented viruses. Segmented negative-sense RNA viruses are further subdivided into families that contain two single-stranded genome fragments such as the *Arenaviridae* (e.g., LASV), three fragments such as the order *Bunyavirals* (formerly the family *Bunyaviridae*, e.g., CCHFV), or multiple fragments such as the *Orthomyxoviridae* (e.g., influenza). Examples of families of non-segmented negative-sense RNA viruses with a single-stranded RNA genome (*Mononegavirales*) include the *Filoviridae* (e.g., EBOV), the *Paramyxoviridae* (e.g., Nipah virus (NiV)), and the *Pneumoviridae* (e.g., RSV). 

Prevention and treatment of infection with negative-sense RNA viruses remain challenging. The 2014 Ebola virus disease (EVD) outbreak in West Africa caused approximately 28,000 cases and 11,310 deaths and is a sober reminder of an unmet medical need [2]. Intensive research efforts during that outbreak led to the discovery and development of several vaccine and drug candidates. Promising experimental vaccines against EBOV are based on the envelope glycoprotein as the antigenic target [3]. Monoclonal antibodies and nucleotide analogue inhibitors represent investigational therapies. ZMapp, a cocktail of three monoclonal antibodies that target the EBOV glycoprotein [4], was shown to reverse EVD in non-human primates [5]. However, results from a randomized clinical trial were inconclusive [6]. Like glycoprotein-based vaccines, the antibody cocktail is also not effective against all ebolavirus species [7,8]. In contrast, several nucleotide analogues show antiviral activity against different ebolavirus species and also other negative- and positive-sense RNA viruses [9,10,11,12,13,14,15,16,17]. 

**Figure 1:**
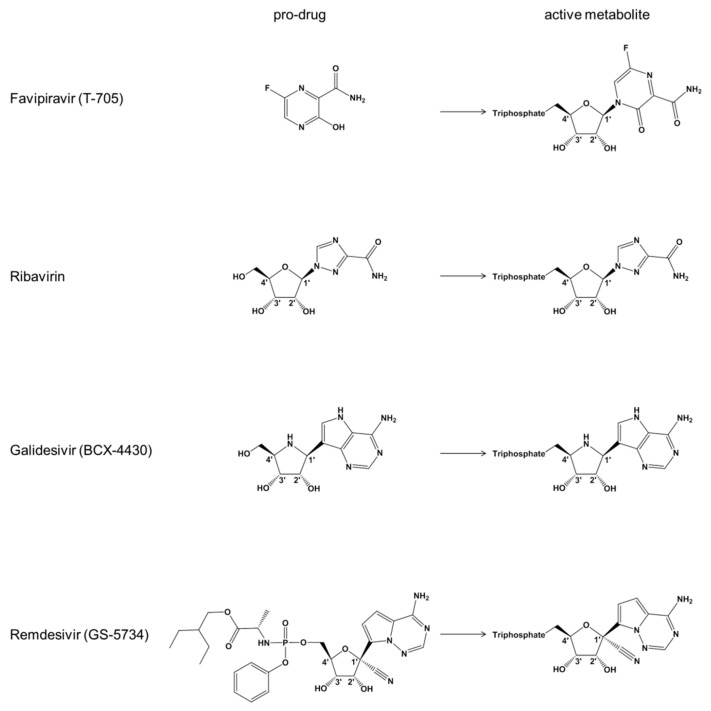
Chemical structures of nucleotide substrate analogues.

Favipiravir (T-705) is a nucleoside precursor that has been approved for treatment of pandemic influenza in Japan [14,18,19]. Intracellular phosphoribosylation yields a triphosphate form (T-705-RTP) that is accommodated by the viral RNA-dependent RNA polymerase (RdRp) (Figure 1) [20]. Experiments with recombinant influenza RdRp show ambiguous base-pairing with both cytidine and uridine, which can lead to lethal mutagenesis [21]. Incorporation of the T-705-RTP into the growing RNA chain can also cause moderate inhibition of RNA synthesis. Favipiravir is active against a large panel of RNA viruses in vitro, including EBOV [12,22,23]. The clinical evaluation has been challenging and results of a multicenter non-randomized trial were largely inconclusive [24]. The structure of the base moiety and proposed mechanism of action are reminiscent of ribavirin that has been used for over a decade to treat infection with the hepatitis C virus (HCV) (Figure 1) [25,26,27,28]. A structurally distinct broad-spectrum antiviral agent that is also active against EBOV in vitro is galidesivir (BCX4430) [11]. Galidesivir is an adenosine-like compound with a nitrogen-substituted sugar ring that likewise requires intracellular activation to the TP form (Figure 1). Biochemical experiments show that this compound serves as a substrate for the RdRp of HCV and causes chain termination following its incorporation [11]. 

Remdesivir (GS-5734) is a phosphoramidate prodrug of a 1′-cyano-substitued adenosine analogue that inhibits ebolaviruses with half maximal effective concentrations (EC_50_) in the submicromolar range [9,10], which is considerably lower than the values reported for favipiravir or galidesivir [11,13,29,30]. The triphosphate form of remdesivir (remdesivir-TP) was shown to inhibit the RSV RdRp surrogate for EBOV RdRp [10]. No significant inhibition was seen with human RNA Pol II and human mitochondrial RNA polymerase (h-mtRNAP) [10]. The biochemical data obtained with purified recombinant RSV RdRp and a recent study with NiV RdRp point to delayed chain termination as a possible mechanism of action [10,31]. Delayed chain termination refers to inhibition of RNA synthesis a few residues downstream of the incorporated inhibitor. However, the inhibition results have yet to be translated in quantitative terms and data with recombinant EBOV RdRp are lacking. RSV, NiV, and EBOV are non-segmented viruses that share similar requirements for RNA synthesis [32,33]. RdRp activity of RSV and NiV requires the multifunctional L protein and the phosphoprotein or P protein [31,34,35]. While the L protein contains the polymerase active site, the P protein is necessary to form an active complex [36]. We have recently expressed active EBOV RdRp that contains the L protein in complex with viral protein 35 (VP35) [33], which is the functional equivalent of P proteins [37]. In this study, we utilized the EBOV RdRp complex to study the mechanism of action of remdesivir. We demonstrate that incorporation of the nucleotide analogue at position i causes delayed chain termination predominantly at position i+5.

## 2. Materials and Methods 

### 2.1. Chemicals 

All RNA primers and templates used in this study were purchased from Dharmacon (Lafayette, CO, USA). 2′C-methyl-ATP and remdesivir-TP were chemically synthesized by Gilead Sciences (Foster City, CA, USA). Ara-ATP was purchased from TriLink (San Diego, CA, USA). NTPs were purchased from GE Healthcare (Cranbury, NJ, USA).

### 2.2. Protein Expression and Purification 

The pFastBac-1 (Invitrogen, Burlington, ON, Canada) plasmid with the codon-optimized synthetic DNA sequences (GenScript, Piscataway, NJ, USA) coding for human mitochondrial DNA-dependent RNA polymerase (h-mtRNAP, NP_005026.3) or viral protein complexes of EBOV (L: AKG65102 and vp35: AKG65095) and RSV (L: AAA84898 and P: AAB59853) RdRp was used as a starting material for protein expression in insect cells (Sf9, Invitrogen, Burlington, ON, Canada). h-mtRNAP construct was designed based on the work by Smidansky et al., 2011 [38] with the following modification: strep- and 8x-histidine tags were added to the N-terminus of the expressed protein. We employed the MultiBac (Geneva Biotech, Indianapolis, IN, USA) system for protein production in insect cells (Sf9, Invitrogen, Burlington, ON, Canada) according to protocols provided by Drs. Garzoni, Bieniossek, and Berger [39,40]. h-mtRNAP and viral protein complexes were purified using the strep- or his-tag-affinity chromatography, respectively, according to the manufacturer’s specifications (IBA, Goettingen, Germany), and Thermo Scientific, Rockford, IL, USA, respectively). The identity of the purified h-mtRNAP was confirmed by mass spectrometry (MS) analysis (Dr. Jack Moore, Alberta Proteomics and Mass Spectrometry, Edmonton, AB, Canada).

### 2.3. Data Acquisition, Quantification, and Analysis 

Data acquisition and quantification were also done as previously reported by us [33]. To account for potential batch-to-batch variations, multiple preparations of EBOV RdRp were used during data acquisition for this study. The amount of EBOV RdRp used in the RNA synthesis assay was optimized such that incorporation of [α-^32^P]-GTP (PerkinElmer, Boston, MA, USA) would reach its maximum after 30 min. The amount of RSV and h-mtRNAP was optimized to have the similar apparent activity as EBOV RdRp by testing various amounts of enzymes. This involved employing low-micromolar concentrations of NTP substrates in RNA synthesis reactions containing h-mtRNAP. RNA synthesis assay consisted of mixing (final concentrations) Tris-HCl (pH 8, 25 mM), RNA primer (200 μM), RNA template (1 μM), [α-^32^P]-GTP (0.1 μM), various concentrations and combinations of NTP and NTP analogues, and EBOV RdRp (1–2 μL) on ice. Note that DNA templates were used in reactions containing h-mtRNAP. The reaction mixtures (10 μL) were incubated for 10 min at 30 °C followed by the addition of 5 μL of MgCl_2_ (5 mM). The reactions were stopped after 30 min by the addition of 15 μL of formamide/EDTA (50 mM) mixture and incubated at 95 °C for 10 min. The 3 μL reaction samples were subjected to denaturing 8 M urea 15% polyacrylamide gel electrophoresis to resolve products of RNA synthesis followed by signal quantification (ImageQuant 5.2, GE Healthcare Bio-Sciences, Uppsala, Sweeden) through phosphorimaging (Typhoon TRIO variable mode imager, GE Healthcare Bio-Sciences, Uppsala, Sweeden). Incorporated nucleotide product fraction was plotted versus nucleotide substrate concentrations and fitted to the Michaelis–Menten equation using GraphPad Prism 7.0 (GraphPad Software, Inc., San Diego, CA, USA).

## 3. Results

### 3.1. Competition between Remdesivir-TP and ATP 

**Figure 2:**
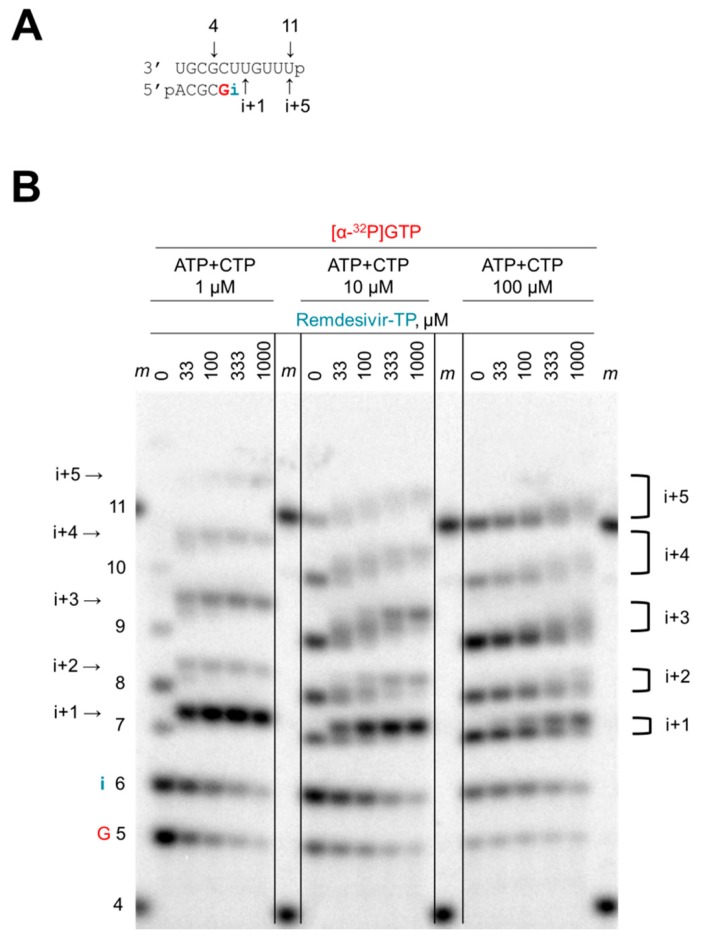
Competition between ATP and remdesivir-TP. (**A**) RNA substrate used in the reaction. Template and primer were both phosphorylated (p) at their 5′-ends. G indicates incorporation of the radiolabeled nucleotide opposite template position 5. Position i allows incorporation of AMP or remdesivir-MP. (**B**) RNA synthesis was monitored with purified Ebola virus (EBOV) RdRp in the presence of [α-^32^P]GTP, three different concentrations of the competing ATP+CTP (1 μM, 10 μM, 100 μM), and increasing concentrations of remdesivir-TP (0–1000 μM). The brackets indicate heterogeneous products containing either AMP or remdesivir-MP, or more complex mixtures due to multiple incorporation sites. Length markers (*m*) represent primer 4 and template 11.

A short model primer/template can serve as a substrate for EBOV RdRp and other viral RdRp enzymes (Figure 2A) [33,35]. This system mimics a random elongation complex that allows the evaluation of nucleotide analogue inhibitors. To elucidate the mechanism of action of remdesivir, we initially assessed whether remdesivir-TP was able to compete with its natural counterpart ATP (Figure 2B). Extension of the primer was monitored with [α-^32^P]GTP that serves as the first substrate in the reaction. The next site of incorporation, referred to as i, allows binding of ATP or the inhibitor remdesivir-TP. Full-length product formation requires the simultaneous presence of ATP and CTP. RNA synthesis was analyzed with three different concentrations of the natural substrates ATP and CTP (1 μM, 10 μM, and 100 μM) (Figure 2B). Increasing concentrations of the inhibitor at each of the three substrate concentrations yielded changes in product patterns that become evident at position i+1. The particular template allows ATP or remdesivir-TP binding at both positions i and i+1. The slower migrating band at position i+1 is indicative of the inhibitor’s incorporation. Most significant differences are seen with the lowest concentration of ATP (Figure 2B, left panel). Higher concentrations of ATP promote synthesis of faster migrating products that do not contain the nucleotide analogue (Figure 2B, middle and right panels). These gradual changes demonstrate competition between ATP and remdesivir-TP. Subsequent RNA products between positions i+2 and i+5 show even more complex shifts as a consequence of multiple competition events. However, a significant reduction in RNA synthesis is not evident under these conditions. We therefore employed a systematic, quantitative approach to evaluate the inhibitory effects of remdesivir-TP.

### 3.2. Selectivity Measurements 

**Figure 3:**
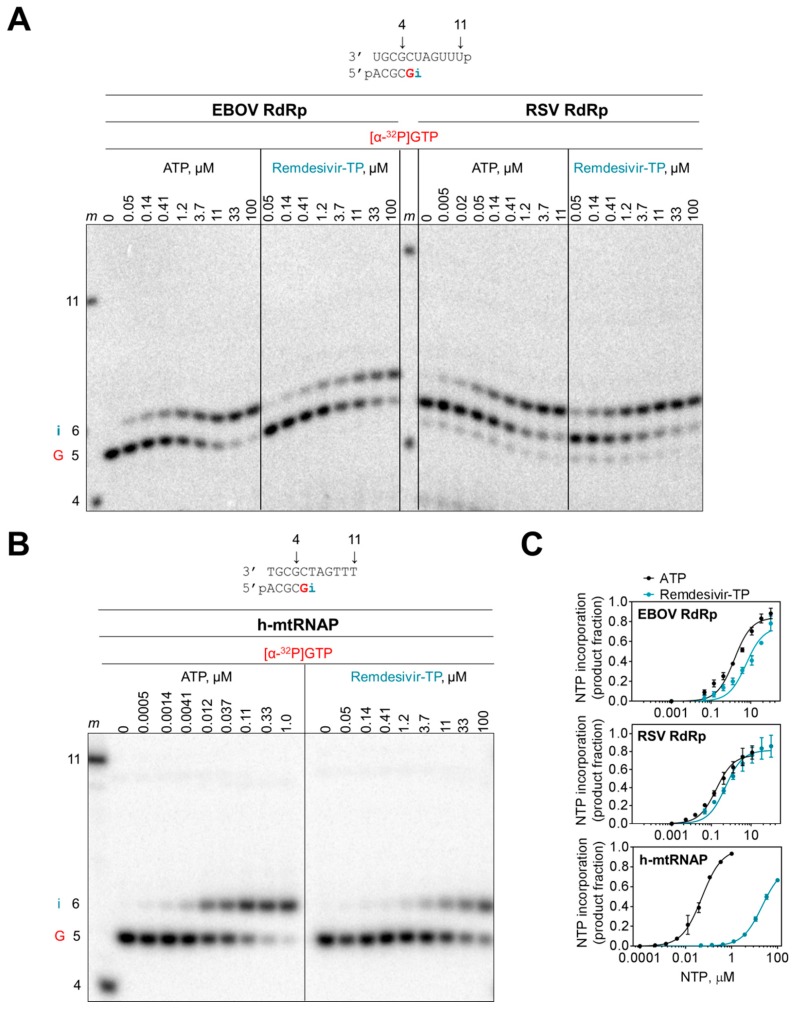
Selective incorporation of remdesivir-MP. (**A**) Efficiency of nucleotide incorporation was studied with purified EBOV RdRp and RSV RdRp complexes. Template and primer were both phosphorylated (p) at their 5′-ends. RNA synthesis was monitored in the presence of [α-^32^P]GTP and increasing concentrations of ATP and remdesivir-TP. **G** indicates incorporation of the radiolabeled nucleotide opposite template position 5. Position **i** allows incorporation of AMP or remdesivir-MP. Length markers (*m*) represent primer 4 and template 11. (**B**) Incorporation of AMP and remdesivir-MP by h-mtRNAP. (**C**) Graphic representation of data shown in (**A**) and (**B**).

We measured the efficiency of incorporation of the monophosphate (MP) AMP and remdesivir-MP at position i with a modified template that does not support base-pairing of adenosine analogues at position i+1. This template allowed us to study single nucleotide incorporations with both EBOV and RSV enzyme complexes (Figure 3A). Increasing the concentration of ATP and remdesivir-TP increased the efficiency of nucleotide incorporation at position i. The data provided kinetic parameters *V*_max_ and *K*_m_ (Table 1). The ratio of *V*_max_/*K*_m_ is a measure for the efficiency of AMP or remdesivir-MP incorporation and the ratio of *V*_max_/*K*_m_ (AMP) over *V*_max_/*K*_m_ (remdesivir-MP) defines the selectivity. Selectivity values of ~4 for EBOV RdRp and ~3 for RSV RdRp demonstrate that remdesivir-TP is almost as efficiently used as ATP. In contrast, human mitochondrial RNA polymerase (h-mtRNAP) discriminates against the inhibitor (Figure 3B,C). The high selectivity value of ~500 shows that incorporation of the inhibitor is inefficient (Table 1).

### 3.3. Chain Termination

**Figure 4:**
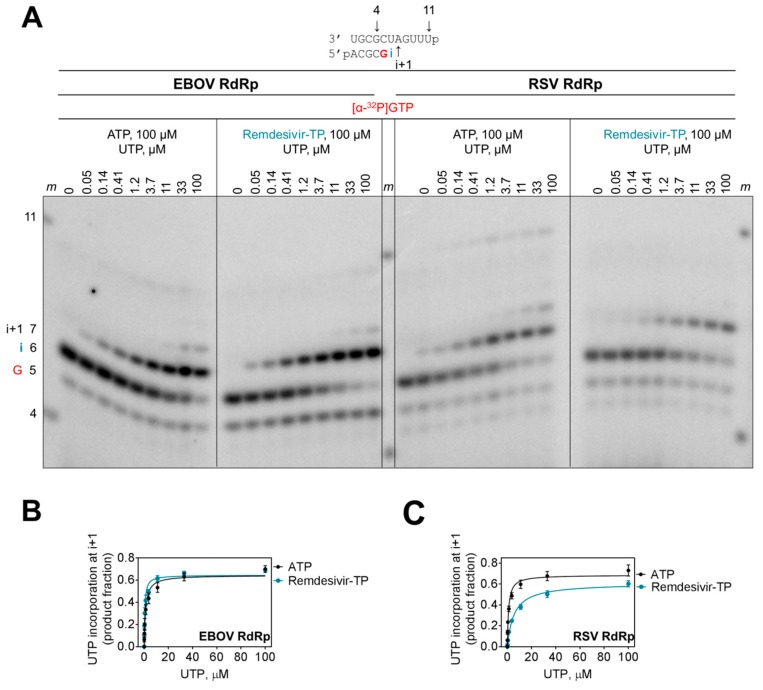
Effect of remdesivir-MP on the efficiency of the next nucleotide incorporation event. (**A**) RNA synthesis was monitored with purified EBOV RdRp and RSV RdRp complexes in the presence of [α-^32^P]GTP, ATP, or remdesivir-TP, respectively, and increasing concentrations of UTP. G indicates incorporation of the radiolabeled nucleotide, i indicates incorporation of AMP or remdesivir-MP, and i+1 indicates incorporation of UMP. High concentrations of UTP also promote UMP misincorporation at the following position. (**B**,**C**) Graphic representation of data shown in (**A**).

The incorporated nucleotide analogue inhibitor can potentially affect incorporation of the next nucleotide. However, the presence of a 3′-hydroxyl group in remdesivir-TP would theoretically allow the nucleophilic attack on the α-phosphate of an incoming nucleotide. To quantify a possible kinetic effect of remdesivir on subsequent incorporation events, we terminated the primer with AMP and remdesivir-MP and measured rates of incorporation of the natural UTP substrate at position i+1 (Figure 4A). For EBOV RdRp, both primers were utilized with the same efficiency (Figure 4B). In contrast, the results with RSV RdRp show that the rate of UMP incorporation is ~6-fold reduced when the 3′-end of the primer contains remdesivir-MP (Figure 4C, Table 2). This difference is primarily driven by a higher K_m_ value. The maximum rate of product formation (V_max_) is almost identical at saturating concentrations of substrate. Hence, the obstacle can be overcome and chain termination is unlikely a possible mechanism of action for both enzymes. At most, the RSV enzyme seems to pause following incorporation of remdesivir-MP.

### 3.4. Delayed Chain Termination 

**Figure 5:**
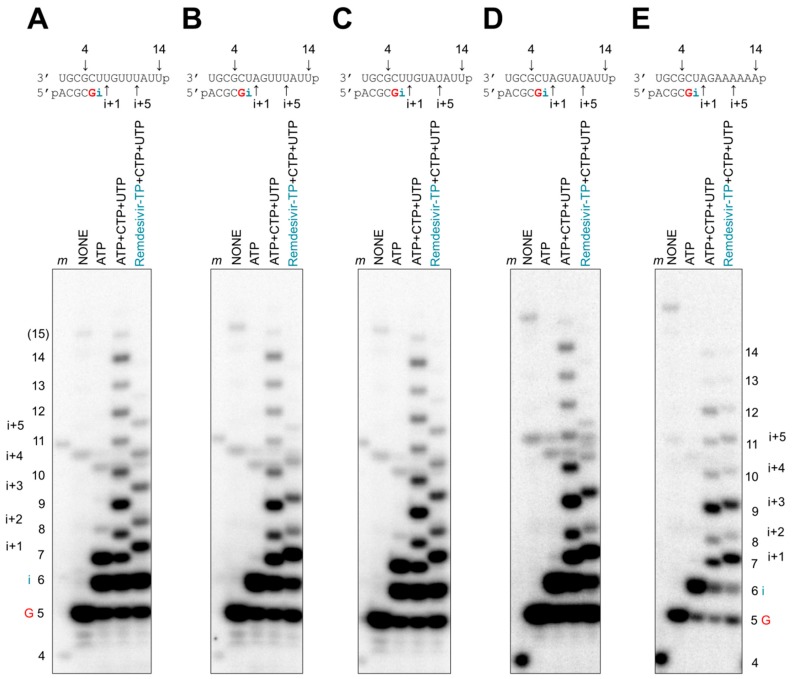
Patterns of delayed chain termination with remdesivir-TPs. (**A**–**E**, top panels) RNA synthesis was studied with recombinant EBOV RdRp on longer templates with 14 nucleotides. (**A**–**E**, bottom panels) Product formation was monitored in the presence of [α-^32^P]GTP and various combinations of NTPs (100 μM) and remdesivir-TP (100 μM). In reactions containing remdesivir-TP, incorporation of i+5 is the longest significant product. The 15-nt RNA product (labeled 15) indicates minor reaction products corresponding to the length of the template plus one nucleotide that is likely generated in a template-independent manner.

We next studied a potential distant effect of the incorporated remdesivir-MP. To this end, we devised longer templates that allowed synthesis of a 14-mer full-length product (Figure 5). To reduce the number of incorporation sites for the inhibitor, we also changed the sequence context in a systematic manner. Adenosine analogues can be incorporated in the following three blocks: (1) i, i+1; (2) i+3, i+4, i+5; and (3) i+7, i+8 (Figure 5A). The control reaction in the presence of all four natural nucleotides yields the expected 14-mer full-length product. A faint band represents a longer 15-mer product that is likely generated in a template-independent manner. RNA synthesis in the absence of ATP and presence of remdesivir-TP is terminated at position i+5, which corresponds to a truncated 11-mer product. This result points to delayed chain termination as a potential mechanism of action. To assess whether delayed chain termination requires consecutive sites of inhibition, we devised templates that were mutated at position i+1, i+4, and at both i+1 and i+4, respectively. In each of these cases, we observed termination of RNA synthesis at position i+5 with only subtle differences in the overall patterns (Figure 5B–D). Hence, incorporation of remdesivir-MP at position i, i+3, and i+5 is sufficient to cause inhibition (Figure 5D). Consecutive sites of inhibitor incorporation are not required. Whether i+3 and i+5 are also necessary for inhibition could not be answered. We devised a template that contained only a single incorporation site at position i; however, overall RNA product formation is here decreased and prematurely terminated even in the absence of inhibitor (Figure 5E). A direct comparison of longer products generated in the absence and presence of inhibitor is therefore not possible. In general, it has been challenging to identify templates with a single uridine that gives rise to sufficient yields of the full-length product. 

**Figure 6:**
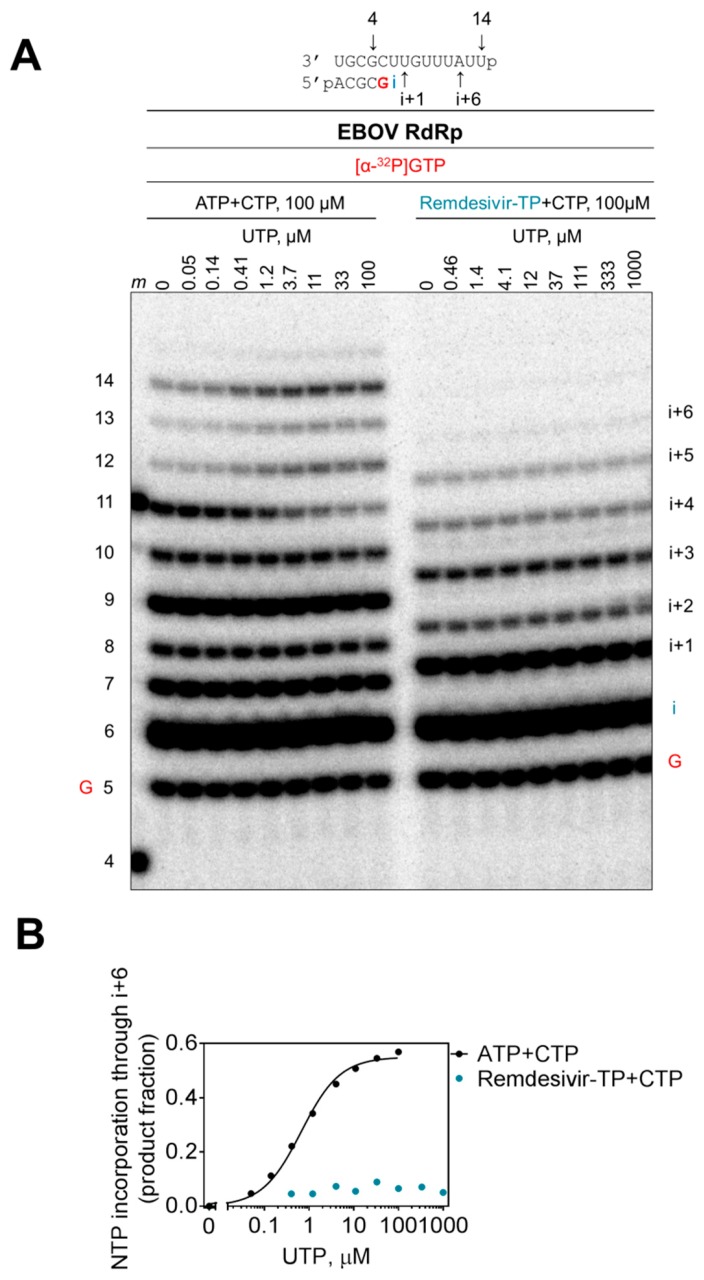
Effective delayed chain termination of RNA synthesis by remdesivir. (**A**) Remdesivir-MP-dependent delayed chain termination of RNA synthesis was studied with purified EBOV RdRp. RNA synthesis was monitored in the presence of [α-^32^P]GTP, CTP, and ATP or remdesivir-TP, supplemented with increasing concentrations of UTP for incorporation at position i+6, representing a 12-nt RNA product labeled 12. (**B**) Graphic representation of data shown in (**A**).

We also studied whether delayed chain termination at position i+5 can be overcome with higher concentrations of the natural nucleotide substrate at position i+6 (Figure 6). Dose–response experiments with concentrations of up to 1 mM of UTP show enhanced product formation in the presence of all nucleotides and absence of remdesivir-TP (Figure 6A, left panel). Product formation plateaus at ~10 μM UTP (Figure 6B). In contrast, no further increase in product formation is seen in reactions with inhibitor (Figure 6A, right panel and Figure 6B). Hence, delayed chain termination is not overcome by higher substrate concentrations.

### 3.5. Susceptibility to Other Nucleotide Analogues 

**Figure 7:**
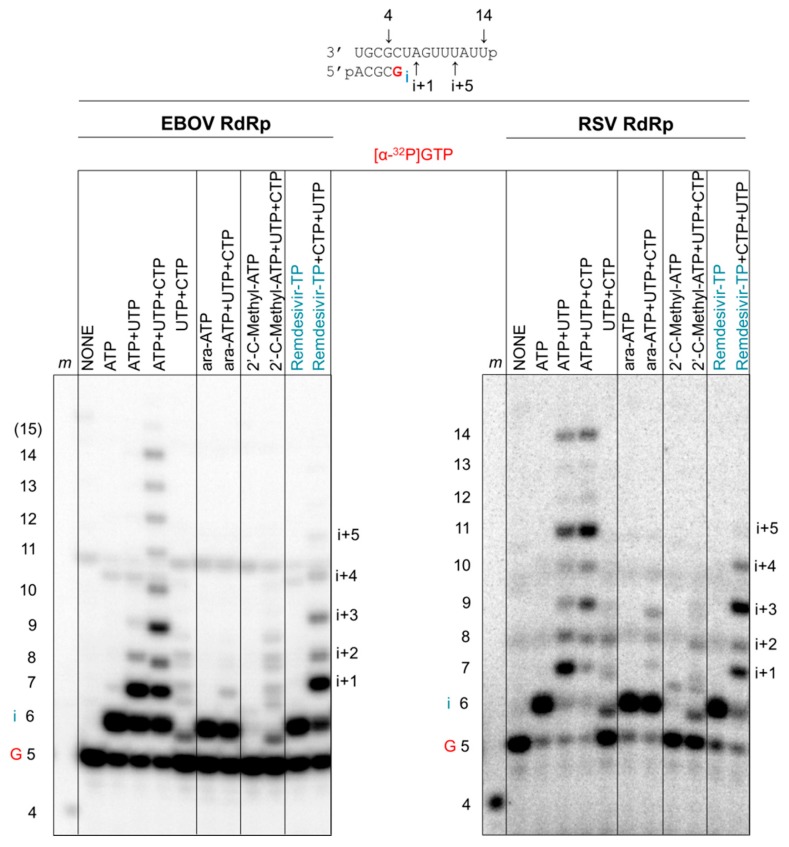
Inhibition of RNA synthesis with different nucleotide analogue inhibitors. RNA synthesis was monitored with EBOV RdRp and RSV RdRp in the presence of [α-^32^P]-GTP and various combinations of 100 μM NTP and 100 μM NTP substrate analogues. The presence of ATP, UTP, and CTP allows full-length product formation up to position 14. The presence of UTP and CTP provides a control for mis-incorporations. Incorporated ara-AMP acts as a chain terminator, 2′-C-methyl-ATP is not incorporated, and remdesivir-MP shows delayed chain termination at position i+5. EBOV RdRp and RSV RdRp exhibit differences in patterns of RNA synthesis and its inhibition by NTP substrate analogues.

We finally compared the inhibition pattern of remdesivir-TP with ara-ATP and 2′C-methyl-ATP (Figure 7). Ara-CTP was previously shown to act as a chain terminator for several RdRp enzymes derived from both negative- and positive-sense RNA viruses [33]. 2′C-methylated compounds are validated chain terminators for HCV RdRp and other positive-sense RNA viruses [41,42,43]. A previous study showed no significant antiviral effects of 2′C-methylated compounds in mini-genome replicons of EBOV [44]. EBOV RdRp (Figure 7, left panel) and RSV RdRp (Figure 7, right panel) both yield the expected full-length 14-mer product in the presence of all four NTPs. In agreement with our previous study [33], ara-ATP is utilized as a substrate and causes chain termination with both enzymes. In contrast, 2′C-methyl-ATP is not utilized as a substrate. Neither EBOV RdRp nor RSV RdRp incorporates the inhibitor at relatively high concentrations of 100 μM. The data are consistent with previous modeling studies that point to a possible steric clash between the 2′C-methyl modification and residues near the active site of EBOV RdRp and RSV RdRp [9]. As discussed, remdesivir-MP is incorporated at position i and causes delayed chain termination at position i+5 with EBOV RdRp and RSV RdRp. Overall, the data demonstrate a specific pattern of inhibition with remdesivir-MP that is common to both polymerase complexes.

## 4. Discussion

Remdesivir or GS-5734 is a nucleotide analogue prodrug that is currently being evaluated in clinical trials for the treatment of EVD [9,45]. Preclinical studies have shown that remdesivir exhibits potent antiviral activity against multiple ebolavirus species [10,16]. Increased intracellular levels of NTP pools correlated with increased antiviral activity, suggesting that the corresponding triphosphate is the active form of the inhibitor and the viral RdRp is the target [10]. Here we confirmed this assumption and demonstrated that remdesivir-TP is a substrate for the purified EBOV RdRp complex. Remdesivir-TP is an adenosine analog and therefore competes with ATP for incorporation. However, the incorporated inhibitor does not act as a chain terminator. Inhibition of RNA synthesis is seen predominantly at position i+5. Despite subtle sequence-dependent effects, RNA synthesis is commonly terminated at this point. Increasing the concentration of the next nucleotide does not overcome this effect. It is therefore reasonable to conclude that delayed chain termination is a major contributor to the antiviral activity of remdesivir. 

The broad spectrum of antiviral activities associated with remdesivir raises the question of whether delayed chain termination is a unifying mechanism of inhibition. Previous data have shown that the inhibitor is also incorporated by RSV RdRp [10]. The inhibitor caused premature termination before the full-length RNA product was formed. A similar result was obtained with the purified NiV RdRp complex and consecutive sites of incorporation seem to enhance this effect [31]. The collective data are in broad agreement with our study. Our enzyme kinetic evaluation revealed that the RSV RdRp enzyme, but not EBOV RdRp, pauses following incorporation of the inhibitor. Despite these differences, delayed chain termination is irreversible and therefore dominant. For both enzymes, the efficiency of incorporation of remdesivir-MP is only 4-fold (EBOV) and 3-fold (RSV) reduced when compared with its natural counterpart ATP. By comparison, a similar experimental approach revealed higher levels of discrimination when favipiravir-TP was studied with purified influenza A polymerase [21]. Favipiravir is incorporated opposite template C and template U. Discrimination against the inhibitor is here 19-fold (opposite C) and 30-fold (opposite U), respectively [21]. The high rate of incorporation of remdesivir likely contributes to the relatively low EC_50_ values in cell-based assays. Remdesivir inhibits EBOV and RSV replication in cell-based assays with EC_50_ values of ~100 nM [10]. In contrast, human RNA Pol II and h-mtRNAP do not seem to be inhibited in the presence of remdesivir-TP [10]. We measured a ~500-fold reduction in efficiency of incorporation of remdesivir-MP over ATP with purified h-mtRNAP. Although potential other off-target effects may not be excluded, these data are consistent with low cytotoxicities in different cell types. For example, 50% cytotoxic concentrations (CC_50_) of remdesivir are generally >100 µM for various cell types [10]. Favipiravir is efficiently used as a substrate by h-mtRNAP, or POLRMT [46,47]; however, the high rates of incorporation do not translate into efficient inhibition [48]. Low inhibition of the cellular RNA polymerase also correlates in this case with CC_50_ values >100 µM [46].

In conclusion, this study provides evidence to show that delayed chain termination is a plausible mechanism of action of remdesivir against EBOV. Delayed chain termination has been described earlier in the context of inhibition of reverse transcriptase (RT) of the human immunodeficiency virus type 1 (HIV-1) and the hepatitis B virus (HBV) [49,50,51]. A prominent example is the mechanism of action of entecavir [51,52], which is approved for the treatment of infection with HBV. Seifer and colleagues reported that the HBV polymerase is inhibited at positions i+2 and i+3 [51]. Entecavir was also shown to exhibit antiviral activity against HIV [53]. Our previous study with HIV-1 RT revealed that a major contributor to inhibition is delayed chain termination at position i+3 [49]. Footprinting experiments provided evidence to show that the enzyme is not adequately positioned to support binding of the next nucleotide. However, the structural reasons for such enzyme repositioning remain elusive. The inhibitory effect of remdesivir is even more distant, which suggests that delayed chain termination can be caused by different mechanisms. Unfavorable interactions between the incorporated inhibitor and distinct elements of the enzyme as well as structural alterations of the newly synthesized RNA must be considered in this regard. This study warrants further investigation into structures of EBOV, RSV, or NiV RdRp complexes and/or the modified double-stranded RNA to address these questions.

## Figures and Tables

**Table 1:** RNA polymerase selectivity values for remdesivir-TP nucleotide analogue.

RNA Polymerase		EBOV		RSV		h-mtRNAP	
Substrate		ATP	Remdesivir-TP	ATP	Remdesivir-TP	ATP	Remdesivir-TP
*V*_max_*^a^* (product fraction)		0.84 *^d^* ± 0.027 *^e^* (3%) *^f^*	0.75 ± 0.039 (5%)	0.76 ± 0.022 (3%)	0.82 ± 0.027 (3%)	0.98 ± 0.018 (2%)	0.81 ± 0.013 (2%)
*K*_m_*^b^* (μM)		1.5 ± 0.23 (15%)	5.7 ± 1.1 (19%)	0.17 ± 0.023 (14%)	0.50 ± 0.089 (18%)	0.050 ± 0.0037 (7%)	21 ± 0.096 (5%)
*V*_max_/*K*_m_		0.56	0.15	4.5	1.6	19.6	0.039
Selectivity *^c^* (fold)		Ref. *^g^*	3.8	Ref.	2.7	Ref.	508

*^a^V*_max_ is a Michaelis–Menten parameter reflecting the maximal velocity of nucleotide incorporation. *^b^* *K*_m_ is a Michaelis–Menten parameter reflecting the concentration of the nucleotide substrate at which the velocity of nucleotide incorporation is half of *V*_max_. *^c^* Selectivity of a viral RNA polymerase for a nucleotide substrate analogue is calculated as the ratio of the *V*_max_/*K*_m_ values for ATP and remdesivir-TP. *^d^* All reported values have been calculated on the basis of a 9-data point experiment repeated three times (n = 3) for natural ATP substrate and the substrate analogue remdesivir-TP for each of the enzymes. *^e^* Standard error associated with the fit. *^f^* Percent error. *^g^* Reference.

**Table 2:** Inhibitory effect of the incorporated remdesivir-MP on the subsequent nucleotide incorporation.

RNA Polymerase		EBOV		RSV	
Primer 3′-end (base)		A	Remdesivir	A	Remdesivir
Substrate		UTP			
*V*_max_*^a^* (product fraction)		0.64 *^d^* ± 0.020 *^e^* (3%) *^f^*	0.65 ± 0.016 (3%)	0.69 ± 0.017 (3%)	0.61 ± 0.014 (2%)
*K*_m_*^b^* (μM)		1.2 ± 0.18 (15%)	0.51 ± 0.07 (15%)	1.0 ± 0.12 (12%)	5.5 ± 0.49 (9%)
*V*_max_/*K*_m_		0.53	1.3	0.69	0.11
Inhibition *^c^* (fold)		Ref. *^g^*	0.42	Ref.	6.2

*^a^V*_max_ is a Michaelis–Menten parameter reflecting the maximal velocity of nucleotide incorporation. *^b^* *K*_m_ is a Michaelis–Menten parameter reflecting the concentration of the nucleotide substrate at which the velocity of nucleotide incorporation is half of *V*_max_. *^c^* Inhibition of subsequent nucleotide incorporation is calculated as the ratio of the *V*_max_/*K*_m_ values for UTP determined on primers ending with AMP and remdesivir-MP. *^d^* All reported values have been calculated on the basis of a 9-data point experiment repeated three times (n = 3) for each of the enzymes. *^e^* Standard error associated with the fit. *^f^* Percent error. *^g^* Reference.
